# Inhibitory Effects of Genistein on Vascular Smooth Muscle Cell Proliferation Induced by Ox-LDL: Role of BKCa Channels

**DOI:** 10.1155/2020/8895449

**Published:** 2020-12-13

**Authors:** Bing Bai, Nanjuan Lu, Wei Zhang, Jinghan Lin, Tingting Zhao, Shanshan Zhou, Elona Khasanova, Liming Zhang

**Affiliations:** Department of Neurology, The First Affiliated Hospital of Harbin Medical University, 23 Youzheng Street, Harbin, 150001 Heilongjiang Province, China

## Abstract

**Background:**

Oxidized low-density lipoprotein (Ox-LDL) is a crucial pathogenic factor for vascular diseases, which can induce the proliferation of vascular smooth muscle cells (VSMCs). Genistein is the main component of soybean isoflavone. Genistein has a variety of pharmacological properties in the treatment of vascular diseases and a promising clinical application. Large-conductance calcium-activated potassium (BKCa) channels are the primary type of potassium channels in VSMCs, which regulate various biological functions of VSMCs. However, whether genistein exerts an antiproliferation effect on Ox-LDL-stimulated VSMCs remains unclear. The current study is aimed at elucidating the effect of genistein on the Ox-LDL-stimulated proliferation of VSMCs and its possible molecular mechanism, especially the electrophysiological mechanism related to BKCa channels.

**Methods:**

Monoculture VSMC was obtained by an acute enzyme-dispersing method. The proliferation of cells was measured by CCK-8, cell cycle, and proliferating cell nuclear antigen (PCNA) expression. The BKCa whole-cell currents were measured by patch-clamp.

**Results:**

Ox-LDL treatment induced the proliferation of VSMCs, upregulated the BKCa protein expression, and increased the density of BKCa currents, while genistein significantly inhibited these effects caused by Ox-LDL. BKCa channels exerted a regulatory role in the proliferation of VSMCs in response to Ox-LDL. The inhibition of BKCa channels suppressed Ox-LDL-stimulated VSMC proliferation, while the activation of BKCa channels showed the opposite effect. Moreover, genistein suppressed the activity of BKCa, including protein expression and current density in a protein tyrosine kinase- (PTK-) dependent manner.

**Conclusion:**

This study demonstrated that genistein inhibited the Ox-LDL-mediated proliferation of VSMCs by blocking the cell cycle progression; the possible molecular mechanism may be related to PTK-dependent suppression of BKCa channels. Our results provided novel ideas for the application of genistein in the treatment of vascular diseases and proposed a unique insight into the antiproliferative molecular mechanism of genistein.

## 1. Introduction

Atherosclerosis, which is characterized by lipid metabolism disorders, is the leading cause of cardiovascular and cerebrovascular diseases, causing significant mortality worldwide [1]. Vascular smooth muscle cells (VSMCs) are the main cell type in the intimal thickening in atherosclerosis. Recent studies have shown that 70% of cells in atherosclerotic plaques are VSMC-derived [2, 3]. Although the mechanisms of atherosclerosis are complicated, the unrestrained proliferation of VSMCs is of vital importance [4, 5]. Therefore, it is of critical importance to study the proliferation of VSMCs in pathological conditions for the treatment of atherosclerosis and the development of antiatherosclerotic drugs.

Large-conductance calcium-activated potassium (BKCa) channels are widely distributed in various mammalian tissues and cells, especially in VSMC [6]. BKCa channels can be activated by intracellular Ca^2+^ or membrane depolarization, which promotes intracellular K^+^ outward and eventually leads to membrane hyperpolarization [7]. The electrophysiological characteristics of the BKCa channel make it a critical target for VSMC dysfunction. It is widely known that BKCa channels are involved in the regulation of vascular tone, and abnormality of BKCa channels leads to vascular diseases, such as hypertension and atherosclerosis [8–10]. Besides, recent studies revealed that BKCa channels are involved in the proliferation of various cells [11, 12], particularly cancer cells [13, 14]. Although the function of BKCa channels in the proliferation of VSMCs has not been fully characterized until now, in view of the electrophysiological characteristics of BKCa channels, we hypothesized that BKCa channels may play a regulatory role in the proliferation of VSMCs.

Oxidized low-density lipoprotein (Ox-LDL) is an independent risk factor for atherosclerosis. It can promote the occurrence and development of atherosclerosis by inducing the migration and proliferation of VSMCs [15, 16]. VSMC dysfunction, particularly the abnormal proliferation caused by Ox-LDL, has become the focus of frontier research on atherosclerosis. Ox-LDL can regulate the activity of various ion channels in different cells. It activated L-type Ca^2+^ channels in cardiac myocytes and A7r5 smooth muscle-derived cell lines [17, 18]. In endothelial cells, ox-LDL enhanced Kv1.5 protein expression and increased the open-state probability (Po) of BKCa channels, which is closely related to Ox-LDL-mediated endothelial proliferation [19]. Nevertheless, whether BKCa channels are involved in the ox-LDL-mediated proliferation of VSMCs has not been extensively studied.

Genistein is a kind of isoflavone extracted mainly from soybean and widely regarded as a phytoestrogen, with a variety of biological properties, including antiproliferative, anti-inflammatory, and antioxidative properties [20–23]. The WHO-CARDIAC and other extensive clinical studies have shown that genistein has a beneficial effect on the prevention and treatment of vascular diseases [24]. Based on multiple clinical trials of genistein with various diseases, genistein shows low oral bioavailability, but it has favorable absorption properties in the intestine [25]. Recent studies have shown that genistein can modulate the function of different ion channels, such as chloride channels, voltage-gated sodium channels, and potassium channels [26, 27]. However, it is still far from clear whether genistein possesses an antiproliferative activity on Ox-LDL-stimulated VSMCs, and a potential molecular mechanism involves the regulation of BKCa channels. The BKCa channel is the main potassium channel with multiple regulatory functions in VSMCs; however, it is unknown whether the antiproliferative mechanism of genistein involves the regulation of BKCa channels.

In this study, we hypothesized that genistein may display an antiproliferative effect on Ox-LDL-induced VSMCs, and its potential molecular mechanism may involve the regulation of BKCa channels. The cell proliferation was measured by CCK-8, cell cycle distribution, and the expression of proliferating cell nuclear antigen (PCNA). The activity of BKCa channels was evaluated using the patch-clamp assay and Western blotting. Our results indicated that genistein exerted an antiproliferative effect on VSMCs infused with Ox-LDL. The molecular mechanisms of this process are probably related to the regulation of BKCa channels by genistein. Our findings may provide new evidence for the application of genistein in Ox-LDL-mediated VSMC dysfunction and also highlight the regulatory function of BKCa channels.

## 2. Materials and Methods

### 2.1. Cell Isolation and Culture

The rat VSMCs were derived from male Wistar rats. Briefly, male Wistar rats aged 10 weeks with a weight of 200 ± 20 g were euthanized by cervical dislocation. Mesenteric arteries were dissected gently from the abdominal aorta, the fat and connective tissues were carefully separated from the arteries, and the arteries were cut into 1 mm long cylindrical sections. These sections were incubated in solution containing 1.5 mg/mL papain, 1.5 mg/mL dithiothreitol, and 1.5 mg/mL BSA for 20–25 min at 37°C, followed by digestion in 1.0 mg/mL collagenase F, 1.5 mg/mL hyaluronidase, and 1.5 mg/mL BSA for 6-10 min. Papain, dithiothreitol, BSA, collagenase F, and hyaluronidase were purchased from Sigma-Aldrich (St. Louis, MO, USA). After digestion, the single smooth muscle cells were dissociated by gentle trituration with a pipette. Cells were maintained in DMEM with the supplementation of 20% fetal bovine serum (Gibco, USA) and 100 U/mL streptomycin/penicillin (Gibco, USA).

The protocol was approved by the Animal Care and Welfare Committee of the First Affiliated Hospital of Harbin Medical University. The experimental protocols were conducted in accordance with the National Guidelines for the Care and Use of Laboratory Animals.

### 2.2. Cell Counting Kit-8 (CCK-8) Assay

VSMCs (5 × 10^3^ cells/well) were plated in 96-well plates and synchronized by serum starving for 24 h, and then, cells were treated with 30 *μ*mol/L of NS1619 (Sigma-Aldrich, USA), 100 nmol/L of iberiotoxin (IBTX, Sigma-Aldrich, USA), or 50 *μ*mol/L of genistein (Sigma-Aldrich, USA) for 6 h before stimulation with or without 75 *μ*g/mL Ox-LDL (Yiyuan Biotechnologies, Guangzhou, China) [26]. Cell proliferation was quantified by a CCK-8 kit (Dojindo Molecular Technologies, Inc., Kumamoto, Japan).

### 2.3. Cell Cycle Assay

After cell growth upon subconfluence, they were synchronized under a serum-free condition for 24 h. The cells were pretreated with 30 *μ*mol/L of NS1619, 100 nmol/L of IBTX, or 50 *μ*mol/L of genistein for 6 h, followed by coincubation with 75 *μ*g/mL of Ox-LDL for another 72 h. The distribution of the cell cycle was determined by a Cell Cycle Analysis Kit (Beyotime, China) using LSR II flow cytometry (BD Biosciences) and analyzed by ModFit software (Verity Software House, Topsham, ME).

### 2.4. Western Blot Assay

The cells were rinsed with PBS three times and lysed in RIPA buffer (50 mM Tris-HCl pH 7.2, 150 mM NaCl, 1% NP40, 0.1% SDS, 0.5% DOC, 1 mM PMSF, and 25 mM MgCl_2_). The proteins were mixed with 2x Laemmli sample loading buffer (Bio-Rad, USA) and denatured at 95°C for 10 min, then separated using 10% SDS-PAGE gel and transferred to polyvinylidene difluoride membranes (GE Healthcare Life Sciences, USA). After blocking with 5% nonfat milk in TBST (0.1% Tween-20), the membranes were incubated in primary antibody for BKCa (ab9276, 1 : 1000, Abcam, Cambridge, UK) or PCNA (ab29, 1 : 1000, Abcam, Cambridge, UK) at 4°C overnight followed by incubation with IRDye 800CW Goat anti-Mouse IgG (H + L) secondary antibody (926-32210, LI-COR, USA) for two hours. The bands were developed by an Odyssey® infrared scanner (LI-COR, Lincoln, USA). The protein expression levels were normalized as the ratio of band intensity to *β*-actin (ab6276, 1 : 5000, Abcam, Cambridge, UK).

### 2.5. Whole-Cell Patch Recording

Cells were perfused with bath solution containing the following (mM): NaCl 145.0, MgCl_2_ 1.2, CaCl_2_ 1.0, KCl 5.6, glucose 10.0, and HEPES 10.0. The pH of the bath solution was adjusted to 7.4. For recording the BKCa currents, 3 mM 4-aminopyridine (Sigma-Aldrich, USA) was used to block the other outward K^+^ currents. The pipette solution consisted of (mM) KCl 140.0, CaCl_2_ 0.686, MgCl_2_ 1.651, EGTA 1.0, K_2_ATP 2.0, and HEPES 1.0 (pH 7.3). The patch electrode resistance was 4–7 M*Ω* after filling with pipette solution. Whole-cell BKCa currents were activated with 500 ms voltage steps from -40 to +80 mV in 10 mV increments. The currents were recorded at a holding potential of -70 mV. After the currents were stabilized, 50 *μ*mol/L genistein, tyrphostin 23, or daidzein (Sigma-Aldrich, USA) was added into the bath solution, and the change of the currents was recorded accordingly. The current density was presented as current amplitude/cell capacitance (pA/pF). Axopatch 700B Amplifier (Axon Instrument, USA), Signal 3.06 software, and CED Power 1401 A/D interface (Cambridge Electronic Design Limited, Cambridge, UK) were used in this study to record whole-cell BKCa currents.

### 2.6. Statistical Analysis

All experiments were performed in triplicates (*n* = 3), and the values were presented as the mean ± standard deviation (SD). A comparison between multiple groups was conducted by using one-way ANOVA followed by Bonferroni correction. Student's *t*-tests were used to compare data between two groups. A *P* value less than 0.05 was considered statistically significant.

## 3. Results

### 3.1. Effect of Genistein and BKCa on Ox-LDL-Mediated VSMC Proliferation

**Figure 1:**
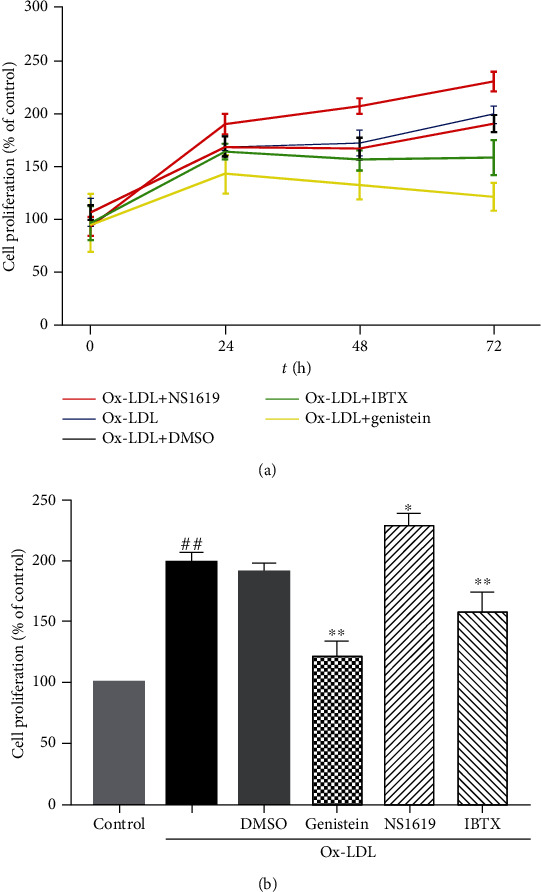
The effect of genistein and BKCa channels on Ox-LDL-induced VSMC proliferation. Cell proliferation was determined by CCK-8. (a) Cell proliferation curves of different treatments. (b) The cell proliferation rate of different groups at 72 h. Ox-LDL increases the proliferation of primary cultured VSMCs. Genistein and IBTX inhibit Ox-LDL-increased proliferation, while NS1619 increases proliferation. DMSO had no significant effect. Results were presented as the mean ± SD from three independent experiments. ^##^*P* < 0.01 vs. control, ^∗^*P* < 0.05 and ^∗∗^*P* < 0.01 vs. Ox-LDL.

Initially, the cytotoxicity of NS1619, IBTX, and genistein in VSMCs was determined by a CCK-8 assay. The concentrations of NS1619, IBTX, and genistein at 30 *μ*mol/L, 100 nmol/L, and 50 *μ*mol/L, respectively, which did not induce cell death were selected for further studies (data not shown). Ox-LDL increased the growth of VSMCs in a time-dependent manner (Figure 1(a)).  Figure 1(b) showed the proliferation rate of VSMCs at 72 h. Ox-LDL significantly increased the proliferation of VSMCs (*P* < 0.01 vs. control). Genistein leads to a significant decrease in cell proliferation (*P* < 0.01 vs. Ox-LDL group). NS1619, the specific BKCa channel activator, increased the proliferation of VSMCs whereas IBTX, the specific BKCa channel blocker, suppressed VSMC proliferation. DMSO had no significant effect on cell proliferation.

**Figure 2:**
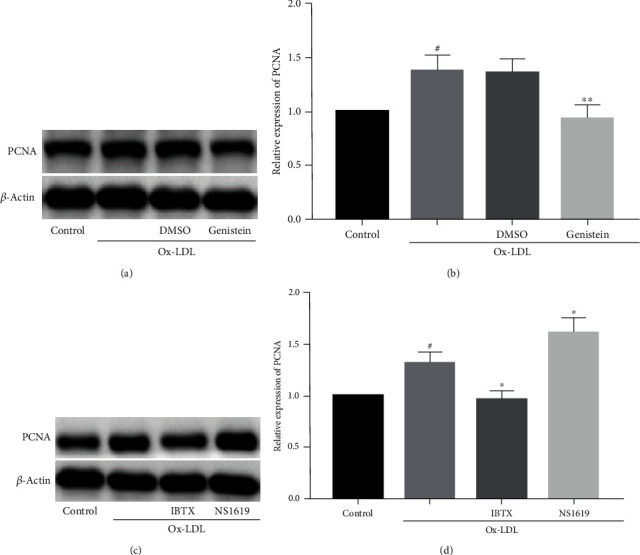
The effect of genistein and BKCa channels on PCNA expression. (a, b) Ox-LDL enhanced the expression of PCNA while genistein reversed this effect. DMSO had no significant effect. (c, d) The expression of PCNA was upregulated by NS1619 and downregulated by IBTX. ^#^*P* < 0.05 vs. control, ^∗^*P* < 0.05 and ^∗∗^*P* < 0.01 vs. Ox-LDL. All the results are expressed as the mean ± SD (*n* = 3 per group).

To further evaluate the proliferation of VSMCs, the expression of proliferating cell nuclear antigen (PCNA) was measured (Figure 2). PCNA is a proliferation marker which is synthesized in the early phase of G0/G1 and S. The expression of PCNA upregulated after stimulation with Ox-LDL. Genistein and IBTX downregulated the high expression of PCNA caused by Ox-LDL. NS1619 upregulated the expression of PCNA in Ox-LDL-mediated VSMCs.

### 3.2. Effect of Genistein and BKCa on Ox-LDL-Mediated Cell Cycle Progression

**Figure 3:**
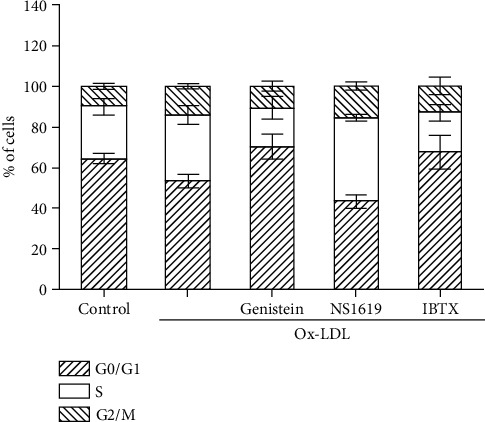
The effect of genistein and BKCa channels on Ox-LDL-induced VSMC cell cycle. Cells were pretreated with genistein, NS1619, or IBTX for 6 h before coincubated with Ox-LDL for another 72 h. The control group is cells without any treatment. The distribution percentages of cells in each phase were presented as the mean ± SD (*n* = 3 per group).

In order to further confirm that genistein and BKCa are involved in the regulation of cell growth, cell cycle analysis was performed. As shown in Figure 3, the percentage of the G0/G1 phase in the Ox-LDL group decreased from 64% to 53% (*P* < 0.01) compared to the control group. Genistein significantly increased the G0/G1 phase to 70% (*P* < 0.01). NS1619 decreased the G0/G1 phase to 43% (*P* < 0.01), while IBTX increased the ratio to 67% (*P* < 0.05). These results further confirmed the modulation role of genistein and BKCa in the proliferation of VSMCs.

### 3.3. Genistein Downregulates Ox-LDL-Enhanced Protein Expression and Current Level of BKCa Channels

**Figure 4:**
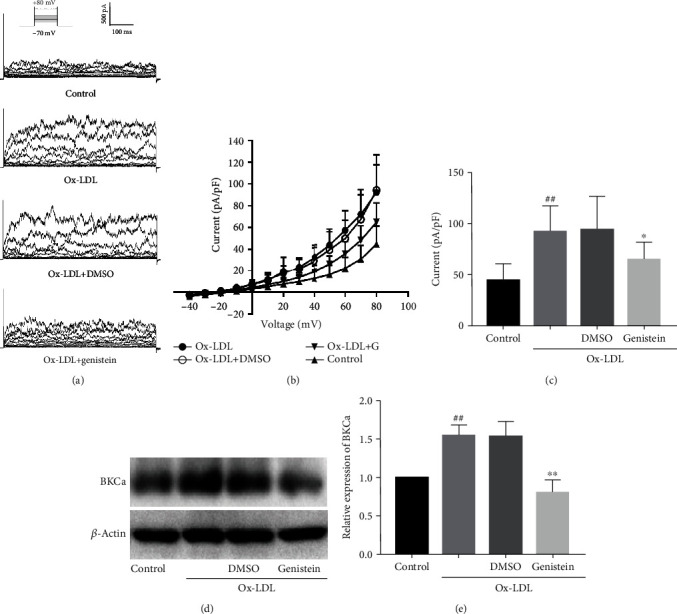
Effect of genistein on protein expression and current level of BKCa channels. (a) Representative BKCa currents. (b) *I*‐*V* curves of BKCa currents. (c) BKCa peak current densities at the test potential of -80 mV. ^##^*P* < 0.01 vs. control, ^∗^*P* < 0.05 vs. Ox-LDL. (d) Western blot analysis and (e) statistical analysis of BKCa channels. ^##^*P* < 0.01 vs. control, ^∗∗^*P* < 0.01 vs. Ox-LDL. All the results are expressed as the mean ± SD (*n* = 3). The data indicated that genistein significantly reversed Ox-LDL-enhanced protein expression and current level of BKCa channels.

After isolation by the enzymatic digestion method, VSMCs were incubated in a culture medium containing 75 *μ*g/mL Ox-LDL for 72 h, and then, single cells were obtained for patch-clamp. BKCa currents were recorded before and after perfusion with genistein (50 *μ*mol/L).  Figure 4(a) shows the representative tracing of whole-cell BKCa currents. BKCa currents were increased by Ox-LDL treatment. The Ox-LDL-enhanced currents were partly reduced by genistein. The voltage-current (*I*‐*V*) curves are displayed in Figure 4(b). As shown in Figure 4(c), at the test potential of -80 mV, BKCa current density of the Ox-LDL group (92.0 ± 24.7 pA/pF, *n* = 15) demonstrated a significant increase compared to the control group (44.3 ± 15.9 pA/pF, *n* = 13, *P* < 0.01). However, after perfusion with genistein, the Ox-LDL-enhanced BKCa current density was decreased to 64.7 ± 17.0 pA/pF (*n* = 17). Peak current densities were lower in the presence of genistein compared to the Ox-LDL group (*P* < 0.05). DMSO caused no significant changes in BKCa currents.

Furthermore, we investigated the effect of genistein on the expression of BKCa. Ox-LDL treatment induced an upexpression of BKCa protein (*P* < 0.01 vs. control) (Figure 4(d)), whereas genistein reversed the upregulation caused by Ox-LDL (*P* < 0.01). DMSO caused no significant differences in BKCa expression. These results suggested that genistein downregulated Ox-LDL-mediated protein expression and current level of BKCa channels.

### 3.4. Effect of Genistein, Tyrphostin 23, and Daidzein on Ox-LDL-Enhanced Protein Expression and Current Level of BKCa Channels

**Figure 5:**
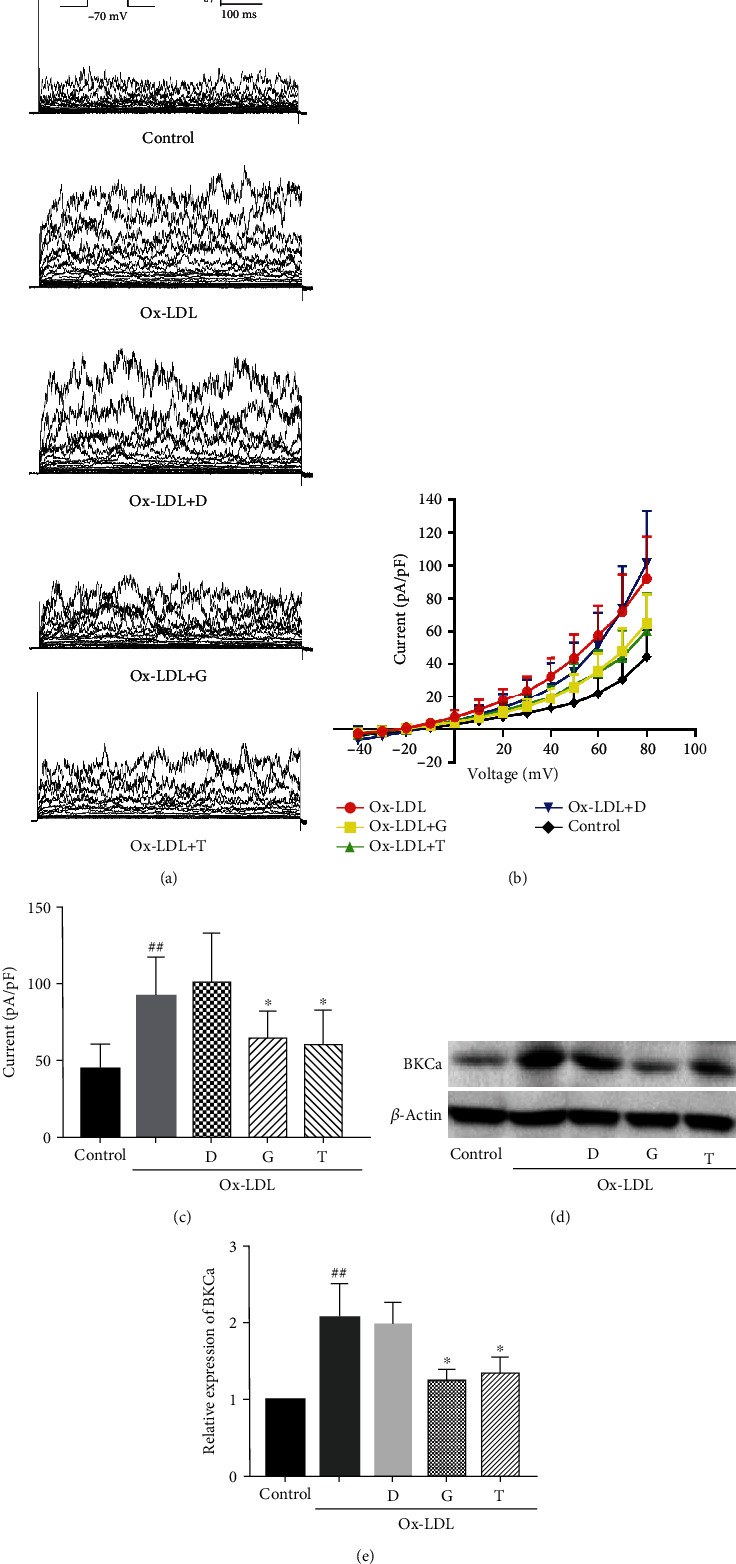
Effect of genistein, tyrphostin 23, and daidzein on protein expression and current level of BKCa channels. (a) Representative BKCa currents. (b) *I*‐*V* curves of BKCa currents. (c) BKCa peak current densities at the test potential of -80 mV. The results indicated that perfusion with genistein and tyrphostin 23 reversed Ox-LDL-enhanced BKCa currents. ^##^*P* < 0.01 vs. control, ^∗^*P* < 0.05 vs. Ox-LDL. (d) Western blot analysis and (e) statistical analysis of BKCa channels. The results indicated that genistein and tyrphostin 23 reversed Ox-LDL-enhanced BKCa protein expression. ^##^*P* < 0.01 vs. control, ^∗^*P* < 0.05 vs. Ox-LDL. All values are expressed as the mean ± SD (*n* = 3). Daidzein had no significant effect on protein expression and current level of BKCa channels. Abbreviations: D: daidzein; G: genistein; T: tyrphostin 23.

VSMCs were cultured with or without 75 *μ*g/mL Ox-LDL for 72 h. Whole-cell BKCa currents were recorded before and after perfusion with genistein (50 *μ*mol/L), tyrphostin 23 (50 *μ*mol/L), or daidzein (50 *μ*mol/L). Figures 5(a) and 5(b) show the representative BKCa current traces and *I*‐*V* relationship curves. As shown in Figure 5(c), at the test potential of -80 mV, Ox-LDL increased current density to 92 ± 25.6 pA/pF (*n* = 13, *P* < 0.01 vs. control); genistein and tyrphostin 23 significantly reduced current density to 64.7 ± 17.6 pA/pF (*n* = 17, *P* < 0.05 vs. Ox-LDL group) and 60.5 ± 22.6 pA/pF (*n* = 13, *P* < 0.05 vs. Ox-LDL group), respectively. Daidzein altered the current density to 101.5 ± 31.9 pA/pF (*n* = 13), which had no significant difference with the Ox-LDL group.

VSMCs were pretreated with genistein (50 *μ*mol/L), tyrphostin 23 (50 *μ*mol/L), or daidzein (50 *μ*mol/L) for 24 h and coincubated with Ox-LDL for another 72 h. As shown in Figures 5(d) and 5(e), genistein and tyrphostin 23 significantly inhibited Ox-LDL-mediated BKCa expression (*P* < 0.05), while daidzein did not affect BKCa expression. Taken together, these results suggested that genistein and tyrphostin 23 rather than daidzein downregulated Ox-LDL-mediated protein expression and current level of BKCa channels.

### 3.5. PTK-Dependent Effects of Genistein on BKCa Currents and Protein Expression

**Figure 6:**
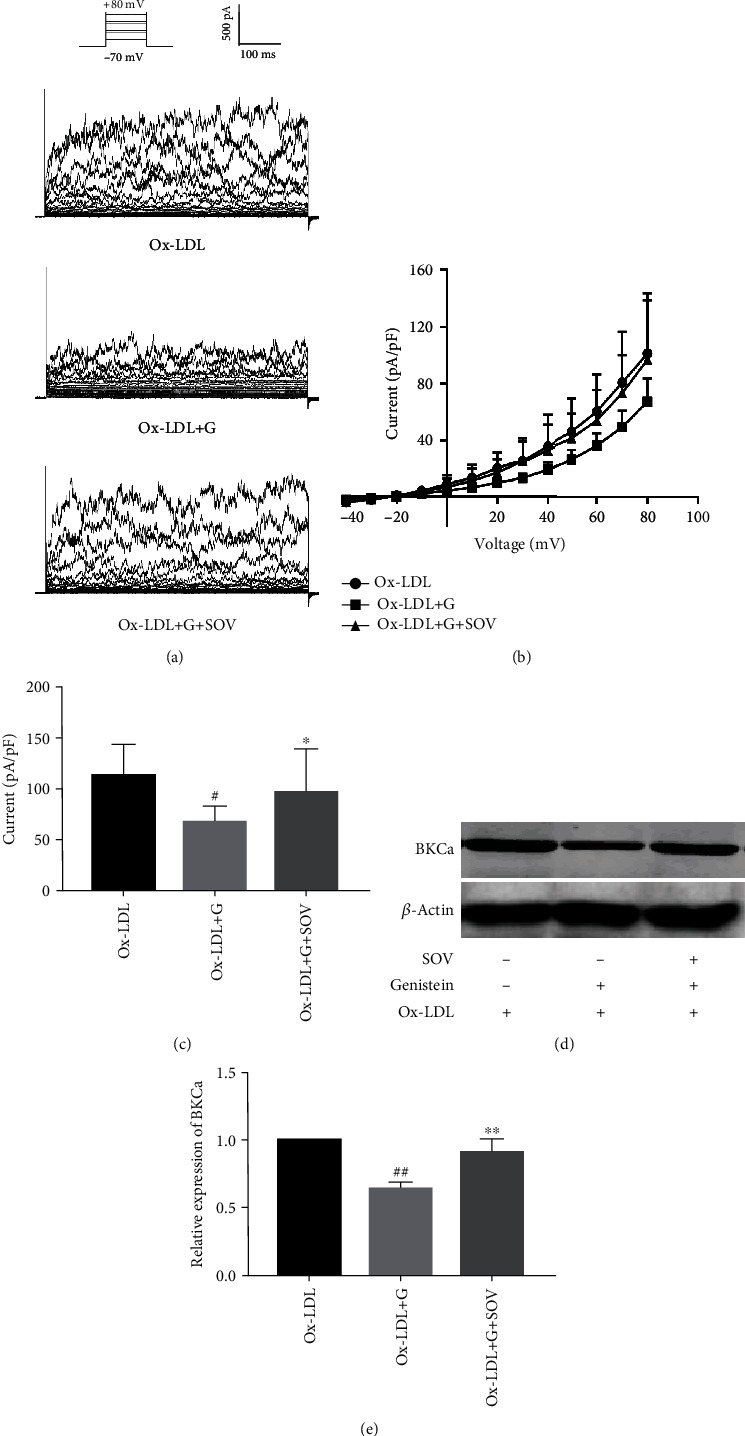
Effects of genistein on BKCa currents and protein expression after preincubation with SOV. (a) Representative BKCa currents recorded in each group. (b) *I*‐*V* curves of BKCa currents. (c) BKCa peak current densities at the test potential of -80 mV. The data indicated that genistein inhibits Ox-LDL-mediated BKCa currents. The inhibitory effects of genistein were reversed by SOV. ^∗^*P* < 0.05 vs. Ox-LDL group. (d) Western blot analysis and (e) statistical analysis of BKCa channels. The result indicated that genistein inhibits the expression of BKCa channels. The inhibitory effects of genistein were reversed by SOV. ^##^*P* < 0.01 vs. Ox-LDL, ^∗∗^*P* < 0.01 vs. Ox-LDL+G. All the results are expressed as the mean ± SD (*n* = 3). Abbreviations: G: genistein; SOV: sodium orthovanadate.

Cells were pretreated with or without 50 mmol/L sodium orthovanadate (SOV) (a protein tyrosine phosphatase inhibitor) for 20 min, and whole-cell BKCa currents were recorded before and after perfusion with genistein (50 *μ*mol/L). Figures 6(a) and 6(b) displayed the representative BKCa current traces and *I*‐*V* relationship curves. As shown in Figure 6(c), at the test potential of -80 mV, the BKCa current density in the Ox-LDL group was 101.1 ± 41.1 pA/pF (*n* = 15); genistein significantly reduced the currents to 67.1 ± 15.7 pA/pF (*n* = 11, *P* < 0.05), while pretreatment with SOV reversed the currents to 96.8 ± 39.0 pA/pF (*n* = 8, *P* < 0.05).

Cells were pretreated with or without SOV (50 mmol/L) for 24 h before coincubation with genistein (50 *μ*mol/L) for another 72 h. Figures 6(d) and 6(e) demonstrated that genistein inhibited Ox-LDL-induced BKCa expression; pretreatment with SOV can reverse the inhibition caused by genistein. These results showed that the inhibition of genistein on BKCa currents and protein expression was related to PTK inhibition.

## 4. Discussion

Genistein is the main component of soybean isoflavone and has multiple pharmacological properties in atherosclerosis prevention [20]. Ox-LDL is involved in almost all pathological stages of atherosclerosis. The abnormal proliferation of VSMCs is a critical pathogenic mechanism of atherosclerosis. In recent years, it has been confirmed that ion channels are closely related to cell proliferation. For instance, CaV 2.1 regulates the proliferation of neural cell [28], and Kv1.3 modulates the proliferation of human VSMC [29]. The BKCa channel is one of the most dominant ion channels in VSMCs and participants in various physiological activities of VSMCs [8]. This study is aimed at determining whether genistein involves in the process of Ox-LDL-mediated VSMC proliferation and revealing the possible role of BKCa channels in this process. Our findings demonstrated that genistein has an antiproliferation effect on Ox-LDL-stimulated VSMCs by inhibition of the protein expression and current density of BKCa channels. These results are of vital importance to the clinical application of genistein for atherosclerosis therapy.

To determine whether the function of BKCa channels is involved in VSMC proliferation in response to Ox-LDL, we utilized the specific channel opener (NS1619) and blocker (IBTX) to regulate the activity of BKCa channels. NS1619 is a selective and reversible activator of BKCa channels and has the potential to modulate cell excitability in neurons and smooth muscle. IBTX is the selective and reversible inhibitor of BKCa channels by binding to a site in the external vestibule of the pore-forming *α*-subunit, whereas NS1619 is the selective agonist to activate the BKCa channel by a direct effect on the Ca+ binding site at the C-terminal end of the *α*-subunit or by the indirect strengthening of the interaction of *β*- to the *α*-binding site [30]. We found that IBTX inhibited the Ox-LDL-mediated VSMC proliferation while NS1619 promoted cell proliferation. Moreover, IBTX induced cell cycle arrest, while NS1619 promoted the course of the cell cycle. These data suggested that the inhibition of BKCa channels reduced Ox-LDL-stimulated VSMC proliferation by preventing cell cycle progression. Our data were consistent with previous reports; inhibition of BKCa channels suppresses endometrial cancer cell proliferation [31]. Previous studies also demonstrated that the inhibition of BKCa channels blocked human bone marrow-derived mesenchymal stem cells in the G0/G1 phase and ultimately suppressed cell growth [32]. Taken together, our results demonstrated that Ox-LDL enhanced the activity of BKCa channels in VSMCs. Moreover, BKCa channels modulated Ox-LDL-induced cell proliferation, indicating that BKCa channels may serve as a potential target for the treatment of VSMC dysfunction.

Genistein inhibited Ox-LDL-enhanced VSMC proliferation by arresting the cell cycle at the G0/G1 phase. Previous studies on the mechanism of genistein inhibition of cell proliferation mainly focused on kinases and transcription factors. Farruggio et al. reported that genistein regulates H9C2 proliferation through the modulation of the Akt, ERK1/2, and p38MAPK signaling pathways [33]. Venza et al. reported that genistein inhibits the proliferation of melanoma cells by preventing PGE2-mediated IL-8 expression [34]. Different from previous studies, our study is aimed at investigating whether BKCa channels participate in the mechanism of genistein inhibition of cell proliferation. Our results indicated that genistein not only reduced the density of BKCa currents but also suppressed the expression of the BKCa protein. As previously described, the inhibition of BKCa channels prevents the abnormal proliferation of VSMCs in response to Ox-LDL. Hence, it is reasonable to ascribe the mechanism of genistein on Ox-LDL-mediated VSMC proliferation, at least partially, to its modulation of BKCa channels.

Genistein, as a phytoestrogen and PTK inhibitor, can affect the activity of various ion channels [35, 36], such as voltage-gated potassium channels (Kv2.1), voltage-gated sodium channels (Nav), and volume-sensitive chloride channels (Cl.vol) [27, 36, 37]. Nevertheless, the effect of genistein on BKCa channels as a phytoestrogen or whether it is PTK-dependent remains unclear. In this study, daidzein is a structural analogue of genistein, and tyrphostin 23 is widely known as another PTK inhibitor. Our research found that genistein and tyrphostin 23 rather than daidzein inhibited Ox-LDL-enhanced BKCa activity, indicating that the two kinds of chemically distinct PTK inhibition factors can regulate the function of BKCa channels. Genistein and daidzein have a similar benzene ring structure, they are the significant isoflavones extracted from soybean, and both function as a phytoestrogen, but daidzein does not demonstrate bioactivity as a PTK inhibitor [38]. In this research, daidzein did not affect BKCa activity, indirectly suggesting the inhibition of BKCa by genistein is associated with its PTK-dependent bioactivity rather than its function as a phytoestrogen. In addition, after pretreatment with sodium orthovanadate (SOV), a protein tyrosine phosphatase inhibitor, the inhibition of BKCa by genistein and tyrphostin 23 was antagonized, which further confirmed that the inhibition of BKCa channels by genistein was PTK-dependent. Our data are consistent with previous studies [39, 40]. Others have shown that PTKs can modulate several ion channels, such as Kir2.1 channels [41], ROMK channels [42], K_ATP_ channels [43], and L-type calcium channels [44]. However, the present research provided the first evidence that genistein suppressed Ox-LDL-enhanced BKCa activity, including protein expression and current density, in primary cultured VSMCs through a PTK-dependent mechanism.

The potential limitation of this study is that our conclusions were based on the research of primary cultured VSMCs, which may not reflect the processes of an intact body. Future investigations are now conducing to validate the effect of genistein on the prevention and treatment of vascular diseases using animal models and *in vivo*.

In summary, the major findings in our study illustrated that (a) Ox-LDL increased the proliferation of primary cultured VSMCs; (b) genistein induced cell cycle arrest at the G0/G1 phase, which suppressed the abnormal proliferation of VSMCs in response to Ox-LDL; (c) the function of BKCa channels can affect the proliferation of VSMCs; and (d) genistein suppressed the activity of BKCa channels, including protein expression and current density, in a PTK-dependent manner. To the best of our knowledge, our study revealed the functional linkage between genistein and BKCa channels in the proliferation of Ox-LDL-stimulated VSMCs for the first time, provided valuable knowledge about the antiproliferative molecular mechanism of genistein, and emphasized the PTK-dependent regulatory mechanisms of genistein on BKCa channels. Nevertheless, future investigations are needed to validate the effect of genistein on the prevention and treatment of vascular diseases using animal models and *in vivo*.

## 5. Conclusions

In summary, genistein inhibited the function of BKCa channels in a PTK-dependent manner by suppressing cell cycle progression and ultimately reducing Ox-LDL-mediated proliferation of VSMCs. Our findings expanded the knowledge regarding the role of genistein in VSMC dysfunction and provided novel insights into the antiproliferative molecular mechanism of genistein in vascular diseases.

## Data Availability

The data used to support the findings of this study are included in the article.
